# Biodegradable Polymer Coated Granular Urea Slows Down N Release Kinetics and Improves Spinach Productivity

**DOI:** 10.3390/polym12112623

**Published:** 2020-11-07

**Authors:** Bilal Beig, Muhammad Bilal Khan Niazi, Zaib Jahan, Salik Javed Kakar, Ghulam Abbas Shah, Muhammad Shahid, Munir Zia, Midrar Ul Haq, Muhammad Imtiaz Rashid

**Affiliations:** 1 Department of Chemical Engineering, School of Chemical and Materials Engineering, National University of Sciences and Technology, Islamabad 24090, Pakistan; bilal_beig@yahoo.com (B.B.); zaibjahan@scme.nust.edu.pk (Z.J.); 2 Department of Healthcare Biotechnology, Atta-ur-Rahman School of Applied Biosciences (ASAB), National University of Sciences and Technology (NUST), Sector H-12, Islamabad 24090, Pakistan; salik.kakar@asab.nust.edu.pk; 3 Department of Agronomy, PMAS-Arid Agriculture University, Murree Road Rawalpindi, Punjab 10370, Pakistan; shahga@uaar.edu.pk; 4 Department of Bioinformatics and Biotechnology, Government College University, Faisalabad 38000, Pakistan; mshahid@gcuf.edu.pk; 5 Research and Development Department, Fauji Fertilizer Company Limited, 156-The Mall, Rawalpindi, Punjab 46300, Pakistan; munirzia@ffc.com.pk (M.Z.); munirzia@gmail.com (M.U.H.); 6 Centre of Excellence in Environmental Studies, King Abdul Aziz University, Jeddah 21589, Saudi Arabia; irmaliks@gmail.com

**Keywords:** controlled nitrogenous fertilizer, polymeric coated urea, biodegradable polymers, granular, release rate

## Abstract

Low nitrogen (N) utilization efficiency due to environmental N losses from fertilizers results in high-cost on-farm production. Urea coating with biodegradable polymers can prevent these losses by controlling the N release of fertilizers. We calculated N release kinetics of coated granular with various biodegradable polymeric materials and its impact on spinach yield and N uptake. Different formulations were used, (i) G-1: 10% starch + 5% polyvinyl alcohol (PVA) + 5% molasses; (ii) G-2: 10% starch + 5% PVA + 5% paraffin wax (PW); (iii) G-3: 5% gelatin + 10% gum arabic + 5% PW; (iv) G-4: 5% molasses + 5% gelatin + 10% gum arabic, to coat urea using a fluidized bed coater. The morphological and X-ray diffraction (XRD) analyses indicated that a uniform coating layer with no new phase formation occurred. In the G-2 treatment, maximum crushing strength (72.9 N) was achieved with a slowed-down N release rate and increased efficiency of 31%. This resulted in increased spinach dry foliage yield (47%), N uptake (60%) and apparent N recovery (ANR: 130%) from G-2 compared to uncoated urea (G-0). Therefore, coating granular urea with biodegradable polymers is a good choice to slower down the N release rate and enhances the crop yield and N utilization efficiency from urea.

## 1. Introduction

Human food demand will increase between 59–98% due to gradual increase in the size of world population from 7.3 to 9.7 billion by 2050, according to Elferink and Schierhorn [1]. This will exert great pressure on farmers to produce more food by either increasing agricultural land for crop production or improving the production potential of existing lands through better management practices. Food production can be increased either by horizontal or vertical expansion. Currently, horizontal expansion is difficult; therefore, vertical expansion is the only option, which means that emphasis must be given to increasing production per unit area. For this purpose, extensive cultivation and excessive dosage of chemical fertilizers, pesticides and other chemicals are being used, which could cause land degradation by depleting soil nutrients and reducing their accessibility to the plants, as well as decrease biodiversity [2,3].

Since the green revolution, a large amount of diverse synthetic fertilizers (nitrogen (N), phosphorous (P) and potassium (K)) have been utilized in agriculture to increase crop productivity [4]. Among others, N scarcity is a major issue severely affecting plant productivity around the globe. Generally, a plant requires 1000 µg N kg^−1^ of dry matter that can be taken up as nitrate (NO_3_^−^-N) and ammonium (NH_4_^+^-N) ions [5,6]. The plant can fulfill this N need by absorbing it from the atmosphere or from indigenous N in the soil. However, atmospheric N is not naturally readily accessible to most crops during their growth [6], and soils in the developing counties (Asia and Africa) are mostly N deficient; therefore, nutrient input rates are higher in these countries [7]. Nitrogenous fertilizer, especially urea, is applied to these N depleted soils to meet the crop N demand. After field application of conventional urea, most of N is wasted in the surrounding through ammonia volatilization, nitrate leaching, runoff and nitrate-denitrification processes [8]. These losses can reach up to 60% worldwide, which increases both fertilizer cost and environmental pollution [9,10]. Consequently, these losses lead to poor plant N use efficiency (NUE) and low yield, thus contributing towards the financial deprivation of the farmers [9,10]. Therefore, there is a need to develop techniques that may help to control or slower down the discharge of N from urea fertilizer and synchronize N availability with crop demand [11].

Urea fertilizers are available in the form of granules and prills. The bigger size of granules compared to prills plays a major function in influencing the physiochemical characteristics of the urea. For instance, prilled urea formulation is more soluble in water than granular due to its smaller dimensions. At the same time, prills are more uniform in shape, whereas urea granules are easier to manage during bagging and transportation, and experience less breakage [12]. To address the issue of higher solubility and volatilization losses, a production methodology of slow-release urea fertilizer was developed in the last few decades [13,14]. These coatings or encapsulations of urea to make it slow a release fertilizer might be an appropriate management strategy that can be used to mitigate NH_3_ volatilization losses, NO_3_^−^ leaching, denitrification and surface runoff [8,9].

Polymeric materials derived from petroleum and plastic products have been used in past decades to produce urea fertilizers with slow release properties [15,16]. The polyethylene [17], polystyrene [18], polyacrylamide [19] and polysulfone[20] materials provided better slow release characteristics than organic or inorganic ones [21,22,23]. The organic polymers were derived from resins and thermoplastic, whereas inorganic coatings were synthesized from sulfur, graphene oxide, gypsum, palygorskite and many other mineral-based materials [21,22,24,25,26]. Nevertheless, these materials are costly and have poor biodegradability, meaning that they can remain present in the soil for a longer duration and disturb the soil functions [13,27,28]. The use of biodegradable materials to coat urea not only reduces the environmental pollution but can also decrease the harmful effects associated with conventional coatings on the proper functioning of soil and plant ecosystems [29,30]. These bio-based materials currently used as coating of fertilizers include waste palm oil [28], animal manure lignin[31], cellulose [32] and starch [33,34,35], which can be used directly or after some modification or mixing with minerals [36,37]. These materials also slowed down N release from urea that could synchronize with crop N demand during the growing season [28,34,38]. Despite some advantages of bio-based/natural product polymer coating, the urea coated by these bio-based materials possesses poor control of nutrient release property. Moreover, their high biodegradability and renewability due to their derivation from natural products is forcing researchers and industrialists to search for innovative and sustainable bio-based materials that could further reduce the cost of coated fertilizer production while providing high efficiency and superior properties of controlled N release from urea fertilizer at a local scale [25,28,39]. Molasses, starch, honeybee wax and gum arabic are bio-based materials with the potential to be used as binder or coating material [35]. Recently, Irfan et al. [40] compared the influence of these bio-based materials on binding and coating efficacy and Zn release efficiency from ZnO coated urea fertilizer. They found that urea granules coated with molasses, paraffin oil and water had better coating efficiency and the best Zn release rate over other materials. Superabsorbent materials are gaining much attraction for controlling the release rate and providing micronutrients to plants and soil [41]. Jimenez-Rosado et al. conducted research by preparing biodegradable superabsorbent bio-plastic (SAB) matrices. The SAB were prepared with soy protein isolate (SPI) as a feed. Zinc was used as a micronutrient, which was incorporated into these bio-plastic matrices to improve product value [42]. Most of the recent studies on natural materials like rice straw and rice husk used one coating material or modified the coating by mixing it with minerals [11,28,31,32,33,34,43,44]. Few studies were also conducted on protein hydrolysate, which has received increasing attention due to its positive effects on plant development. These materials act as plant biostimulants due to their protein nature [45,46,47]. 

Slow-release fertilizer delays N availability from urea after its soil application, which results in delaying plant N uptake and utilization processes [48]. Most of the studies available to date in the literature have only investigated controlled N release kinetics at lab scale in distilled water or soil columns. Costa, Cabral-Albuquerque [49] observed complete coated urea dissolution in distilled water within two minutes. In a very recent study, bio-based material coated urea completed its urea release in water in 80 days [28]. Similarly, Guo, Liu [50] incubated encapsulated urea granules in the soil and observed 10, 15 and 61% release rates of coated urea during 2, 5, and 30 days of incubation, respectively, without any crop. According to Naz and Sulaiman [8], the main future research goal in the field of coating fertilizer would be to increase crop N uptake efficiency from the applied urea fertilizer. Therefore, coating technology should be designed in a way that could help release N or nutrients that would be synchronized with plant N demand [39,51]. To meet this objective, the evaluation of N release from the applied coated fertilizer would be tested at field scale in addition to the lab-scale experimentation. However, to the best of our knowledge, field-scale experimentation lacks evidence to support this objective.

The major objective of this research is to use locally available natural biodegradable polymers for the coating of urea fertilizer. The novelty behind this work is to develop new coating formulations with benign chemicals (with reference to biodegradation and lower cost) and therefore reduces the urea release. The fluidized bed coater (FBC) technique, which is considered the most efficient technique for the production of slow-release fertilizer [52,53], was adopted for the synthesis of coated urea. The coating solution of polymers was initially prepared, and then sprayed on granules with the help of FBC. A UV-Visible spectrophotometer was utilized to evaluate the urea release from coated granules. Scanning electron microscope (SEM), XRD, Fourier transform infrared (FTIR) and crushing strength testing of prepared samples was performed. The lesser objectives are (i) The characterization and N release kinetic study of coated urea with various polymeric materials and (ii) investigate the impact of slow-release polymer-coated urea on the N uptake and spinach yield.

## 2. Material and Methods

### 2.1. Materials

Starch, polyvinyl alcohol, gelatin, gum arabic, paraffin wax, concentrated hydrochloric acid (33.5%) and p-dimethyl amino benzaldehyde of laboratory grade were obtained from Daejung Chemicals & Metals Co., Korea. Molasses was provided by Almoiz Sugar Mills Unit II, Dera Ismail Khan, Pakistan. Commercial grade urea granules were purchased from a local market and contained 46% nitrogen content (Fauji Fertilizer Bin Qasim Limited, Sindh, Pakistan). Urea was sieved to get uniform sized particles of 4 mm. All chemicals were utilized without any further purification.

#### 2.1.1. Preparation of Polymeric Solution

First of all, 10 g of starch was dissolved in 100 mL of de-ionized water preheated at 80 °C for 30 min. Afterward, suggested amounts of PVA and PW were added and mixed with constant stirring at 80 °C for 2 h (Table 1). Consequently, the coating solutions were ready for coating applications. Same method was used for all other compositions, as mentioned in Table 1, including gelatin, gum arabic and molasses. Starch was replaced with gelatin for G-3 and G-4 formulations.

#### 2.1.2. Coating of Urea Fertilizer Using Fluidized Bed Coater

The granular urea was coated using a YC-1000 mini spray granulation unit (developed by Shanghai Pilotech Instrument & Equipment Co., Ltd.). The coating material was introduced from the bottom through a spray nozzle. The granules were fluidized using hot fluidization air with a blower frequency of 45 Hz. The prepared polymeric blend was pumped with the help of small pump at 30 rpm. The atomizer air pressure was maintained at 0.2 MPa by air compressor for spraying of hot solution. The fluidized bed temperature was maintained at 80 °C. The hot solution spraying started when the fluidized bed temperature reached 80 °C. After completion of coating solution, the granules were dried with hot air for 15 min.

#### 2.1.3. Characterization of Formulated Urea Granules

The uncoated and coated urea granules were characterized by using FTIR, SEM, XRD, crushing strength testing and UV-Visible spectrophotometry.

##### Fourier Transform Infrared (FTIR) Spectroscopy

FTIR analysis was done for the qualitative analysis of the uncoated and coated urea granules. This analysis was performed on FTIR PerkinElmer Spectrum 100 spectrometer at wave number range of 4000–500 cm^−1^. The granules were first finely powdered and then used for analysis. Fourier transform infrared (FTIR) spectroscopy was used to examine the presence of different functional groups during the process of polymeric coating.

##### Scanning Electron Microscopy (SEM)

Surface morphology of granular samples was examined by scanning electron microscope (S-4700, Hitachi, Japan). Prior to examination, gold sputtering was carried out on the urea granules using a JEOL JFC-1500 ion sputtering machine. Gold coating was carried out on the granules up to 250 angstroms. Accelerating voltage 20kV was used for the analysis of samples at a magnification of 2000×.

##### X-ray Diffract

XRD analysis was carried out by using STOE Germany. The step size and scan ratio were 0.4 s^−1^ and 1 step^−1^, respectively. The scan angle was kept at 20° to 70°. The wavelength of CuKα radiation was 1.540 Ả. XRD analysis was done to investigate the crystallinity of the coating surfaces formed [54].

##### Crushing Strength

The crushing test of prepared samples was carried out to ensure stability of granules against impact during the synthesis phase until their packaging and transportation. Urea granules start to break and turn into fine powder due to their delicate nature [55]. This powdered urea has no further usage for agricultural purposes. The crushing test was performed on the coated urea granules using ultimate tensile machine of AGS-X. The test was started by applying stress with the help of metal plunger. The amount of stress where the granules cracked was noted. This value is known as a crushing strength [55]. 

##### UV-Visible Spectrophotometer

The urea release rate and efficiency of the synthesized granule with polymeric materials were calculated by the p-methyl amino benzaldehyde method [55]. The experiment started by drawing calibration curve using 99.9% pure urea. Standardized urea solutions were prepared to obtain the slope from the calibration curve. The above standard solution absorbance was measured using UV-Visible spectrophotometry. To calculate the release rate of different formulations (G-0 to G-4), the following procedure was adopted.

#### 2.1.4. Procedure for Release Rate

First, 10 g of sample was put in a 5 Liter beaker with de-ionized water. The beaker was stirred for 15 s before sample collection. Next, 10 mL of liquid was collected at different intervals of 3, 6, 9, 12, 15, 30, 60 and 120 min from beaker. After this 1 mL of HCl (1:1) and 5 mL of p-dimethyl amino benzaldehyde solutions were added with addition of de-ionized water to make the volume to 50 mL. At last, the flask was slightly shaken and its absorbance was measured using 418 nm wavelength. Finally, this absorbance was used to calculate the concentration of the sample using Equation (1). After that, concentrations of coated and uncoated urea granules at 30 min were used for the calculation of efficiency using Equation (2) [56].

(1) Urea(ppm)=(Absorbance−Y.Intercept)÷(Slopefromcalibrationcurve)

(2) $$\mathrm{Efficiency}\left(\%\right)=\frac{C_{U}-C_{CU}}{C_{U}}\times 100$$

where, *C_U_* and *C_CU_* are the urea concentrations(ppm) in the uncoated and coated formulations, respectively, at 30 min.

##### Release Kinetics

The kinetic study of urea release was measured to investigate the mechanism of N release from urea when applied to soil in the presence of water. Urea release rate data was used to find the mechanism of N release. The obtained data was converted into fractional urea release. After that, three modified equations (hyperbola, Schwartz, and Schwartz and Sinclair) were applied to the fractional urea release. All the equations describe the diffusion phenomena for the transportation of nutrient when samples interacted with the water. [56,57].

The dissolution rate of the entire combination was applied to the following formulas:

The modified hyperbola formula is shown below [56,57]:

(3) $$\mathrm{Qt}=\frac{at}{1+bt}$$

First order kinetic parameters are linked with small values of time. On the other hand, increase of time changes the reaction order from one to zero.

Schwartz and Sinclair’s formula is shown below [56,57]:

(4) $$\mathrm{Qt}=\left(1-e^{-bt}\right)$$

The modified Schwartz formula is represented below [56,57]:

(5) $$\mathrm{Qt}=a\left(1-e^{-bt}\right)$$

Whereas, *Q_t_* is denoted as the fractional release of urea at time interval *t*, while *a* and *b* are known as release constants for different active agents.

### 2.2. Pot Experiment

A standardized pot testing was carried out with sandy clay loam soil at the Fauji Fertilizer Company Research Center, Faisalabad, Pakistan. Soil was taken from the research center, where maize was previously grown. This soil was classified as Typic Camborthids (Govt. of Pakistan, 1974). Afterward, sieving was done with 2 mm mesh for the removal of all the debris. After sieving, each pot with 30 cm diameter was filled with 13 kg of soil. In total, six treatments, including a control (untreated), each with four replicates (6 × 4), were allocated in 24 earthen pots. The experiment was carried out in a completely randomized design (CRD). Treatments were: (i) C: control (untreated); (ii) G-0: uncoated granular urea; (iii) G-1: granular urea coated with 10% starch + 5% polyvinyl alcohol (PVA) + 5% molasses; (iv) G-2: granular urea coated with 10% starch + 5% PVA + 5% paraffin wax (PW); (v) G3: granular urea coated with 5% gelatin + 10% gum arabic + 5% PW; (vi) G4: granular urea coated with 5% molasses + 5% gelatin + 10% gum arabic. Coated and uncoated fertilizer treatments were used with the dose of 100 kg N ha^−1^ in four splits, i.e., before sowing and after the first, second and third harvest of the crop. Phosphorus and potassium based fertilizers were also added immediately before sowing the crop with the rate of 75:100 kg PK ha^−1^. A basal dose of each fertilizer was mixed in the top 5 cm of the soil layer. After treatment application, seeds of a local cultivar of spinach were hand sown at a rate of 0.177 g pot^−1^. To meet ambient conditions for the growth of spinach plants, pots were kept in an open space. Regular irrigation of pots was done to keep the moisture level 60% using a watering can with extreme care, while the increase in soil water content was measured using moisture meter (FY-901, Hangzhou FCJ I&E Co., Ltd., China). 

#### 2.2.1. Soil Sampling and Analysis

Initial (before treatment application) and final (after the last harvest of spinach crop) soil sampling was done from a soil depth of 0-15 cm with the help of a hand augur. Four soil samples were initially taken before pot filling. Finally, four samples of soil were collected from random spots from each pot and finally mixed to get a composite mixture. Soil mixture of each fertilizer treatment was analyzed for pH, electrical conductivity (EC), total organic carbon (TOC), dissolved organic carbon (DOC), mineral N and plant available P and K. Soil pH was measured from a 1:2.5 soil and water extract by using a pH meter (inoLab pH Level 1, WTW GmbH & Co. KG, Germany). Afterward, soil EC was measured from the same mixture using an EC meter (DDS-12DW). Soil TOC content was measured through the wet oxidation method [58]. Soil DOC was determined according to the procedure described in Shah et al. (2019). Mineral nitrogen (ammonium-N (NH_4_^+^-N) and nitrate-N (NO_3_^−^-N) were measured using the ammonium bicarbonate-diethylene tri amine penta acetic acid (AB-DTPA) method. Soil available P and K were measured by procedures described in [59]. 

#### 2.2.2. Plant Analysis

Spinach was harvested four times during the whole pot testing experiment, i.e., 65, 102, 151 and 184 days after sowing. Leaf chlorophyll content was measured immediately before each harvest by using a SPAD chlorophyll meter [60]. Fresh foliage yield was measured immediately after each harvest. Dry foliage yield was determined by weighing the foliage of all plants in each pot after complete drying of the samples at 70 °C for 48 h until a constant weight of the samples was obtained. Afterward, each dried sample was ground to pass through a 1 mm sieve and analyzed for total N content by the Kjeldahl digestion method.

#### 2.2.3. Apparent N Recovery

Apparent nitrogen recovery (ANR) of fertilizer was calculated as the difference in nitrogen uptake between plots receiving nitrogen and plots without nitrogen. ANR from coated and uncoated urea treatments was calculated using the equation below:

(6) $$\mathrm{Apparent}\;\mathrm{Nitrogen}\;\mathrm{Recovery}\left(\mathrm{ANR}\right)\left(\%\right)=\frac{\left(N_{FT}\times DM_{FT})-(N_{C}\times DM_{C}\right)}{N_{applied}}$$

where *N_FT_* is *N* content (g N (100 gDM)^−1^) of spinach in fertilized treatment, and *DM_FT_* is spinach dry foliage yield (kg ha^−1^) in fertilized treatment. *N_C_* is *N* content (g N (100 gDM)^−1^) of spinach in the control (unfertilized) treatment and *DM_C_* is spinach dry foliage yield (kg ha^−1^) in the control (unfertilized) treatment. *N_applied_* is the total *N* application rate (kg ha^−1^) per treatment.

#### 2.2.4. Statistical Analysis

The formulation effects were subjected to univariate analysis by using SPSS Statistics version 19. The major effects of fertilizer were tested by using analysis of variance (ANOVA). The means of all treatments were compared at 5% probability level. When treatment effects were significant, then multiple comparisons among treatments were analyzed by Tukey’s HSD test.

## 3. Result

### 3.1. Effect of Coating on Surface Morphology

**Figure 1:**
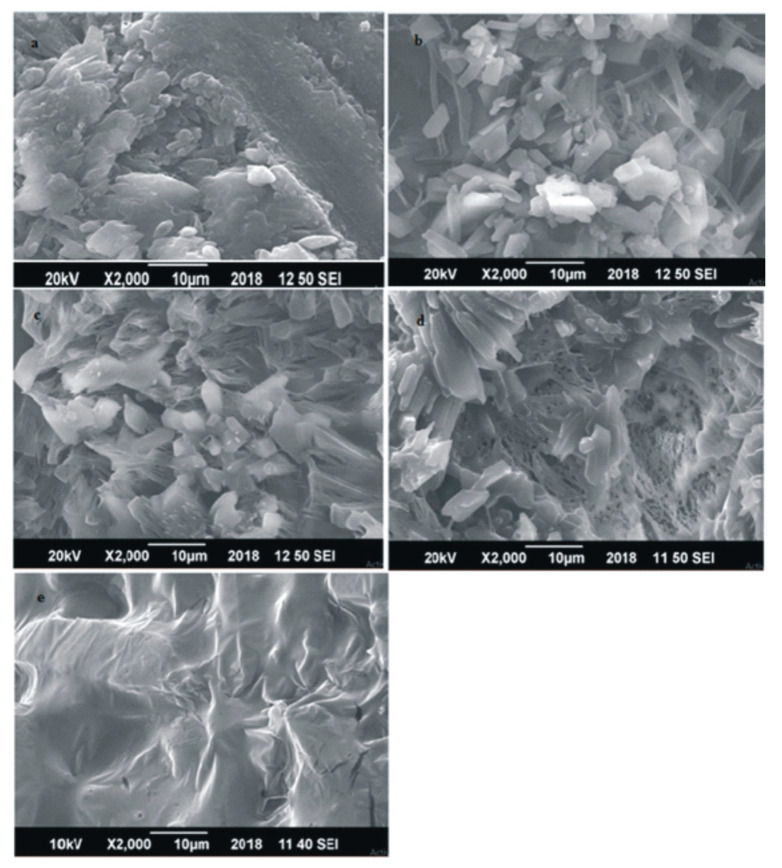
SEM micrographs of granular urea: (**a**) G-0: Uncoated, and coated with (**b**) G-1: 10% starch + 5% polyvinyl alcohol (PVA) + 5% molasses; (**c**) G-2: 10% starch + 5% PVA + 5% paraffin wax (PW); (**d**) G-3: 5% gelatin + 10% gum arabic + 5% PW; (**e**) G-4: 5% molasses + 5% gelatin + 10% gum arabic.

High-magnification SEM micrographs illustrated the surface morphology of uncoated and coated granular urea fertilizers (Figure 1a–e). The SEM micrograph of the uncoated urea granules showed an agglomerated structure with an uneven surface and rough patches. As a result of coating, SEM micrographs of coated urea revealed that the outer layer of coated granules was denser and smoother as compared to uncoated granules.

### 3.2. FTIR

**Figure 2:**
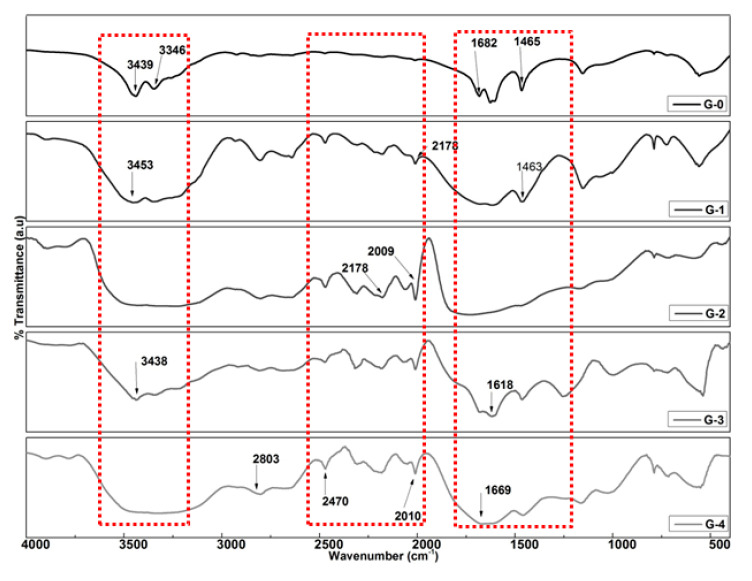
FTIR spectra of uncoated and coated formulations.

FTIR spectra of different formulations are presented in Figure 2. The shoulder peak at 3439 and 3346 cm^−1^ confirmed presence of N-H group in G-0 [58,59]. A sharp peak of carbonyl (CO) was absorbed at 1662 cm^−1^ [60]. Furthermore, a strong intensity of NH and vibrational stretching of O=C-NH_2_ was detected at 1465 cm^−1^ [61]. Band stretching at 1154 cm^−1^ observed due to presence of –C-O-C group [55]. The stretching vibration due to C-H was observed at 1154 cm^−1^ [62]. The G-1 and G-2 treatments showed a sharp vibrational peak at 3453 cm^−1^ due to NH_2_ group. Peaks observed at 2178 and 2009 cm^−1^ are attributed toward the presence of alkyne (C≡C) compounds in both G-1 and G-2 coating materials. For G-3 and G-4 spectra, additional peaks were observed at 2100 cm^−1^ of nitrile (C≡N) and aldehyde (=C-H) at 2803 cm^−1^.

### 3.3. XRD

**Figure 3:**
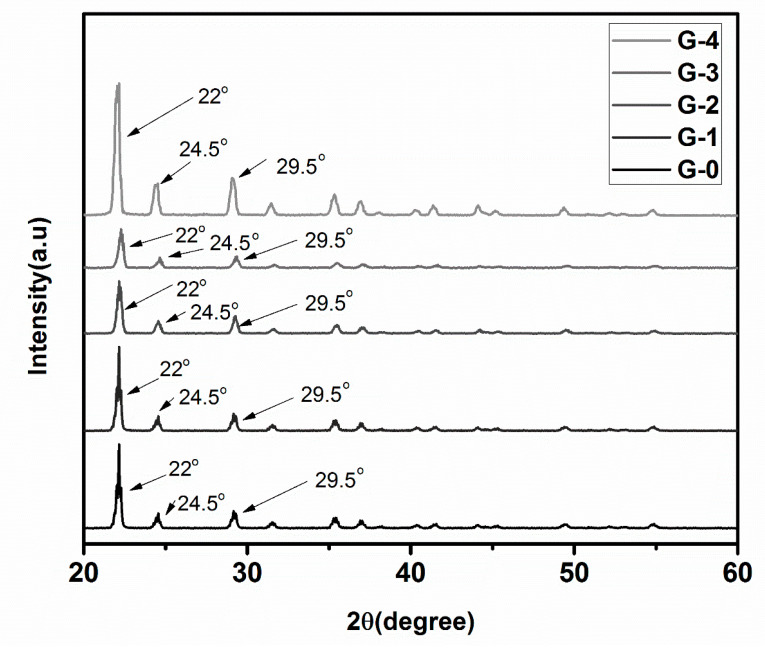
X-ray diffraction (XRD) spectrum of uncoated and coated granular.

The XRD analysis for all formulations is presented in Figure 3. In uncoated urea granule (G-0), the characteristic diffraction peaks were observed at 22°, 24.5°, 29.5° and 35°. More dominant peaks were present in the range of 22°to 25°. All coated formulations showed similar sharp peaks in the range of 22° to 25°[63].

### 3.4. Effect of Coating on the Crushing Strength

**Figure 4:**
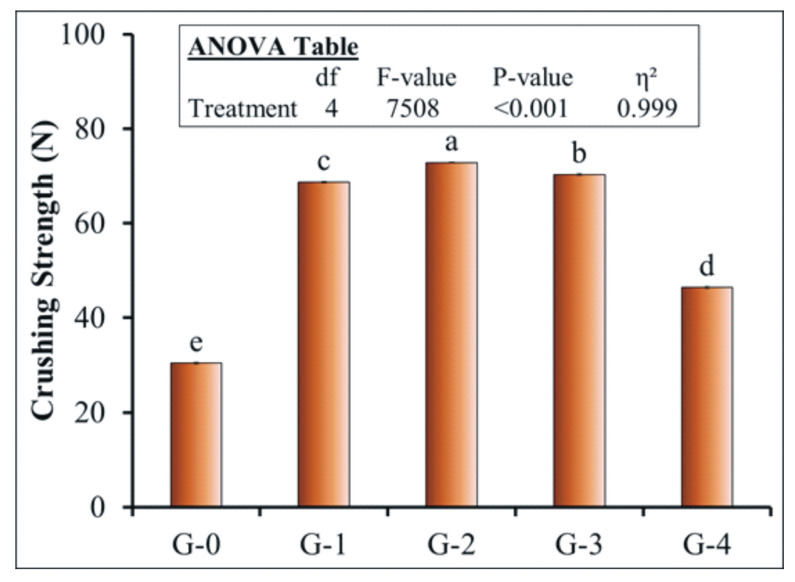
Effect of coating on crushing strength of granular urea. Symbols used for fertilizer treatments can be seen in the caption of Figure 1. Small letters on bars indicate the difference among treatments at 5% probability level. Error bars show the standard error of the mean (n = 4). Insets in the figure represent outcomes of the analysis of variance (ANOVA).

The crushing strength results are presented in Figure 4. The G-0 treatment (uncoated urea) was crushed at a force of 30.55 N. The highest crushing strength was observed in the granules of the G-2 treatment (Figure 4**)**. G-4 showed the lowest value of crushing strength. On the other hand, the G-3 treatment showed comparable value with G-2. In the former treatment, gelatin and gum arabic also formed an excellent coating layer on granules in the presence of paraffin wax, which enhanced its crushing strength [62] similarly to the G-2 treatment (Figure 4).

### 3.5. Effect of Coating on the Rate of Urea Release

**Figure 5:**
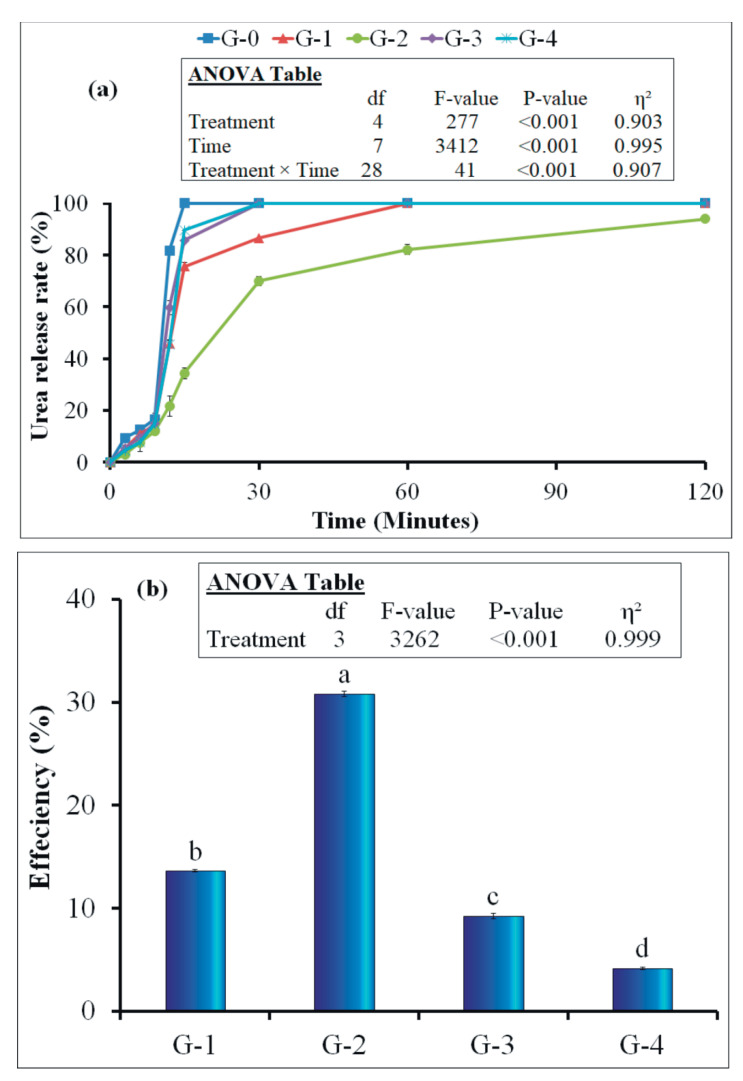
Urea Release rate (**a**) and urea release efficiency (**b**) of granular urea. The symbols used for various formulations can be seen in the caption of Figure 1. The small letters on error bars indicate the difference among treatments at 5% probability level. The error bars indicates standard error of the mean (n = 4). Insets in the figures represent outcomes of the analysis of variance (ANOVA).

The urea release from coated urea granules measured using UV-Visible spectroscopy is shown in Figure 5 [56]. The release results obtained from the test were reported in terms of percentage. The efficiency of different formulations was evaluated at 30 min by using Equation (2).

In the G-0 (uncoated urea) treatment, urea was completely released in water after 15 min and 37 s of immersion. The G-1 treatment released urea completely within 60 min and 45 s. The urea release efficiency in the G-2 treatment was the highest at 30.8%, due to the presence of paraffin wax as a binding agent. Consequently, the G-2 coating combination released urea completely within 130 min and 45 s. Our findings on the G-2 treatment were in line with a few other studies on biodegradable coating materials, where the researchers found 94% of urea was released after 120 min of its immersion in water [57,62]. On the other hand, the G-3 treatment showed 9.3% urea release efficiency. The G-4 showed the lowest efficiency (4.2%) among all. Hence, this treatment (G-4) released urea completely in water within 30 min and 21 s.

### 3.6. Release Kinetics

The fitting results are displayed in Table 2. The release data were best fitted by using a modified hyperbola formula compared to the other two equations. The fitted data showed very precise values of high correlation constant and was significantly better than modified Schwartz equation, as well as the Schwartz and Sinclair formula.

### 3.7. Pot Experiment

Influence of biodegradable polymeric coated slow release granular urea on soil chemical properties, spinach yield and N uptake.

**Figure 6:**
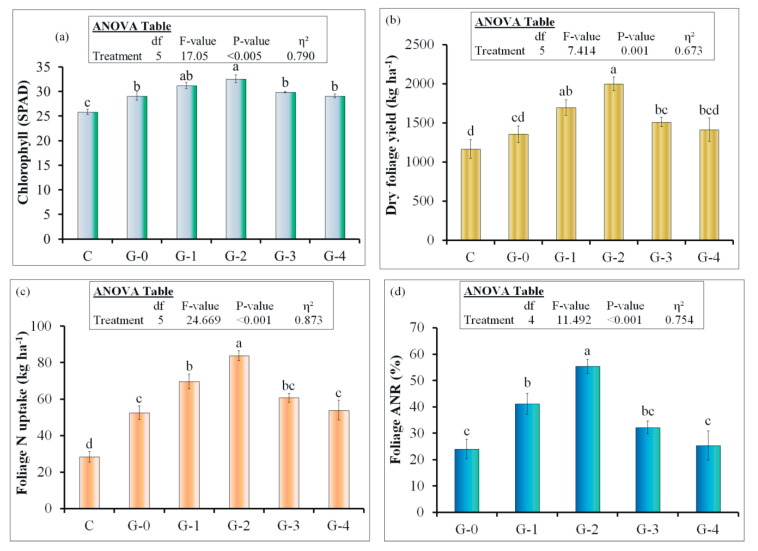
Spinach (**a**) chlorophyll content, (**b**) dry foliage yield, (**c**) N uptake and (**d**) apparent N recovery (ANR) from coated and uncoated urea treatments. Abbreviation for fertilizer treatments can be seen in the caption of Figure 1 and C stands for control (untreated). The small letters on the error bars represent the difference between different treatments at 5% probability level. Error bars show the standard error of the mean (n = 4). Insets in the figures represent outcomes of the analysis of variance (ANOVA).

The soil chemical parameters such as pH, electrical conductivity (EC), dissolved organic carbon (DOC), mineral N, plant-available P and K are presented in Table 3. All coated urea treatments significantly decreased soil pH (7.4 vs. 7.9) in comparison with uncoated urea. However, dissolved organic C was significantly higher in all coated urea treatments than G-0 or the control. Moreover, soil mineral N at the end of the experiment was also higher in all urea coated treatments compared to uncoated urea or the control. The chlorophyll content of the spinach leaves was significantly higher in G-1 and G-2 treatments than uncoated urea (Figure 6a). However, this parameter did not differ significantly among G-0, G-3 and G-4 treatments. Among the coated urea treatments, the highest chlorophyll content was observed in G-2, and the lowest in G-3 and G-4. Moreover, spinach dry matter (DM) yield, N uptake and apparent N recovery from the applied uncoated and coated fertilizers was also the highest in G-2 and lowest in G-4 and G-0 treatments (Figure 6b–d), which was in agreement with chlorophyll content (Figure 6a). Among coated urea treatments, G-2 increased spinach DM yield by 47% (2000 vs. 1357 kg ha^−1^), and in the case of G-1, the increment in this parameter was 25% (1697 vs. 1357 kg ha^−1^) compared to G-0 treatment. However, G-3 and G-4 did not significantly increase this parameter over the G-0 treatment (Figure 6b). This increment in DM yield for G-1 and G-2 treatments was also evident in spinach N uptake (Figure 6c). The latter parameter was 33 and 60% higher in G-1 and G-2 treatments, respectively, compared to uncoated fertilizer treatment (G-0). Similar to DM yield, N uptake in G-3 and G-4 treatments was not different to G-0. Similar to Nitrogen uptake, spinach ANR from coated fertilizer was higher than uncoated urea (Figure 6d).

## 4. Discussion

The uncoated urea surface looked jagged throughout the granule (Figure 1a), with visible pores. This was in line with research by Naz and Sulaiman [64], who also observed similar high porous surface of uncoated urea. The granule surface was gradually coated by coating materials including starch and gum arabic, along with polyvinyl alcohol (PVA) and gelatin as film-forming materials. However, paraffin wax and molasses acted as a binder. The surface of coated urea was observed to be uniform, rigid, dense and water-resistant, as observed by many other studies on coated urea fertilizer with biodegradable polymers [28,57,64]. Among the coated urea treatments, the G-1 treatment had scattered elongated crystals with PVA film behind, as shown in Figure 1b. Moreover, few starch agglomerates were also present, with stacked structure and high heterogeneity in the sample. Alternatively, the G-2 treatment had compacted and non-uniform shape structures over the entire granule, shown in Figure 1c. Its surface area was gradually covered with a layer containing starch and paraffin wax. In this case, the heterogeneity of the sample could be decreased by the paraffin wax. This process was quite visible in the G-1 treatment, where a mixture of starch, PVA and molasses was used as the coating material (Figure 1b). The granule surface of the G-3 treatment was porous throughout the entire surface (Figure 1d). This indicated that a formulation of gum arabic, gelatin and paraffin wax was unable to remove the defects in the urea granule surface, and this was ascribed to poor compatibility of the coating material. The G-4 treatment resulted in a smooth and even coating over the entire granule (Figure 1e). Therefore, this coating formulation is responsible for blocking pores and filling the opening of the pores during the process of surface layer formation [55].

At 3256 cm^−1^, the G-1 treatment showed an OH vibration, which implied that water was absorbed. The alkyne (C≡C) stretching vibration at 2178 and 2009 cm^−1^ can be ascribed to the presence of PVA and starch as coating materials [65]. The carbonyl (CO) peak was observed at 1627 cm^−1^. The sharp peaks appeared at 1465 cm^−1^ was due to presence of amide functional group in urea [65]. The additional peaks of nitrile (C≡N) at 2100 cm^−1^ and aldehyde (=C-H) at 2803 cm^−1^ were due to the presence of gelatin and gum arabic used as coating materials [55,65].

Almost similar pattern was observed in all formulations [56]. The characteristic diffraction peaks of starch were seen at 22°, and 24.5°. Peaks appeared at 22°and 24° were attributed to gelatin and PVA respectively. Few peaks appeared around 40° were due to presence of gum arabica in coating formulation of G-3 and G-4 [35,66].

From urea storage and transportation point of view, fertilizer granules with superior impact resistance are preferred [62]. In present study, the crushing strength of uncoated and different polymer material coated urea treatments were checked using tensile testing machine. The final reading was noted when the granules were completely crushed into fine powder. The results of this test are presented in Figure 4. The G-0 treatment (uncoated urea) was crushed at a force of 30.55 N. The highest crushing strength of G-2 treatment (Figure 4) was due to the presence of active ingredient paraffin wax [65]. The PVA and starch formed a compact film around the urea granule, whereas paraffin wax tightly sealed the whole granule surface and therefore showed the highest strength [63]. It is evident from the results that on replacing the coating material with materials such as gelatin or gum arabic, the urea granules had a considerable impact on the crushing strength. Among all combinations of materials used to coat the urea granules, G-4 showed the lowest value of crushing strength. This might be attributed to the jelly-like formation of coating material in the presence of gelatin and gum arabic [67]. However, in this treatment, molasses was also used as an adhesive binding agent that helped to ease the coating of urea granules. In all coated treatments, the crushing strength was significantly higher than the uncoated urea; hence, all coating materials used in our study enhanced the crushing resistance of the urea granules compared to commercial urea.

The G-0 treatment followed the burst release phenomena due to the absence of coating on it, as observed by Rodríguez-Félix, Pérez-Martínez[68] in their amoxicillin release experiment. Due to the presence of coating, the G-1 treatment released urea completely within 60 min and 45 s. This can be attributed to the water-soluble nature of molasses [40]. Its suppression effect on the release rate was less [69] compared to G-2. The coating layer of starch and PVA bonded with molasses gradually disintegrated and reached a point where the burst release of urea occurred, as was the case for the G-0 Treatment. The highest efficiency of G-2 was ascribed to the low dissolution of paraffin wax as a coating material in water that helped to retard the urea release rate [56,65]. In the aforementioned treatment, starch and PVA formed more reliable coating with wax as compared to molasses. Therefore, such a coating combination has a higher capability to sustain against water than the coating combination containing molasses [69]. Moreover, the wax completely sealed the entire granule, leaving no cracks or pores on the surface, as clearly seen in the SEM image (Figure 1c). The 9.3% efficiency associated with G-3 was due to poor compatibility of gelatin and gum arabic with paraffin wax [67]. In this treatment, the paraffin wax was unable to seal and bind the coating material on the granule surface, which resulted in poor film formation, as was evident from the SEM image (Figure 1d). Therefore, complete urea released in this treatment occurred within 30 min and 10 s. The lowest efficiency (4.2%) among all the treatments of G-4 was explained by the higher solubility of molasses in water [40]. Conversely, the adhesion property of molasses might create a more compact coating layer on the granule surface as molasses bound efficiently with gelatin and gum arabic to form coating with additional levels of compactness (Figure 1e). However, due to the hydrophilic properties of gelatin and molasses, the combination failed to retard the release rate more than 4.2%, at which point the gelatin started to swell and allowed water to penetrate the film, which might dissolve the core [56,65].

Curve fitting technique was used on fractional urea release to investigate the kinetic parameters [57]. The fractional urea release data were best fitted using a modified hyperbola formula with higher adjusted R^2^ values (Table 2). First-order kinetic parameter explained the N release from the coatings composed of matrix [70]. It may be concluded that the modified hyperbola formula is the best model to depict the N release from formulated granules.

Montaño, García-Oliva [71] proposed that the presence of a high quantity of DOC supports the formation of NH_4_^+^-N in the soil and, at the same time, enhances the microbial demand of dissolved organic N and NH_4_^+^-N. By doing so, the DOC favors N recycling in the soil-plant system. This higher DOC in coated urea treatments could be associated with high mineral N in these treatments (Table 3) [71]. The increment in soil mineral N from all coated urea treatments and especially G-2 could be in line with many other studies that found high mineral N content after coated urea application to the soil, despite the fact that they used soil column and laboratory incubation studies without plants [72,73]. The coated urea treatments were capable of delaying the process of urea hydrolysis and, therefore, they slowed the rate of NH_4_ release compared to uncoated urea [73]. On the other hand, rapid hydrolysis took place in the uncoated urea that resulted in faster release of NH_4_. Therefore, in our study, despite the spinach growth in the pots, we observed higher mineral N content in soil of coated urea pot after completion of experiment.

The chlorophyll content in leaves indirectly indicates crop health, and its nutritional status is affected by C and N balance [74]. Therefore, higher chlorophyll content in G-1 and G-2 treatments means that nutrients, especially N, were available for the crop when needed. Since chlorophyll content is closely correlated with the accumulation of N in the plant leaves, it is a more informative parameter for estimating the crop N uptake from the soil [75]. This is in accordance with our laboratory scale and pot experimental data of relatively lower urea release rate (Table 3, Figure 5a), which gave an indication that available N from the soil must synchronize with plant N demand in G-1 and G-2 treatments compared to the uncoated urea, which is also in line with research by Geng, Ma [51].

Among coated urea treatments, G-2 increased spinach DM yield by 47% (2000 vs. 1357 kg ha^−1^). This increment in yield was due to the slow release of urea, which met the plant sequential need. Spinach apparent N recovery (ANR) from coated fertilizer was higher than uncoated urea (Figure 6d). This higher spinach N uptake or ANR in G-2 treatment could be related to delaying the hydrolysis of urea by coating with the mixture of starch, PVA and paraffin wax [33,56]. This delayed hydrolysis synchronized N release from urea fertilizer that matched well with crop N requirements [51]. Therefore, we observed higher spinach DM yield, N uptake and ANR than uncoated urea.

## 5. Conclusions

This study evaluated the use of different biomaterials to coat urea and the influence of coated urea on urea release kinetics in water, soil and N uptake in spinach crops. Our results showed that G-2 treatment, consisting of a combination of starch, PVA and paraffin wax as coating materials, showed excellent urea release efficiency (30.3%), with improved crushing strength (72.97 N). The slower release performance of coated urea granules from this treatment synchronized the N availability from urea granules to the spinach crop demand. This was quite evident from the results of the highest soil mineral N, spinach N uptake and apparent N recovery for G-2 treatment compared to other coated and uncoated urea treatments. Hence, it was concluded that the G-2 coated formulation was best and could be applied to fulfill all necessary needs for slow-release fertilizer, including economic, bio-degradable and environmentally friendly nitrogenous fertilizer requirements. The developed fertilizer formulations should also be tested for crops grown over different soils to observe their agronomic performance. For future perspective, the formulations should also be investigated at different soil moisture contents to evaluate the N release rate and coating layer degradation.

## Figures and Tables

**Table 1:** Composition of biodegradable polymer coatings of granular urea.

Treatment	Starch	PVA *	Molasses	Gelatin	Gum Arabic	Paraffin Wax
	(g/100 g of Urea)					
G-1	10	5	5	-	-	-
G-2	10	5	-	-	-	5
G-3	-	-	-	5	10	5
G-4	-	-	5	5	10	-

* PVA = Polyvinyl alcohol.

**Table 2:** Release kinetics of uncoated and coated granular urea at 25 ºC calculated by using three different model equations.

Name of Model	Treatment	Adjusted R^2^	Value of “a”	Value of “b”	χ^2^
Modified hyperbola	G-0	0.7185	0.0924	0.0731	0.0614
	G-1	0.8136	0.0798	0.0627	0.0366
	G-2	0.9200	0.0327	0.0242	0.0109
	G-3	0.7853	0.0712	0.0546	0.0444
	G-4	0.9116	0.0287	0.0198	0.0129
Schwartz and Sinclair formula	G-0	0.5954	-	0.1583	0.08832
	G-1	0.8282	-	0.0851	0.02908
	G-2	0.8082	-	0.0509	0.02633
	G-3	0.6891	-	0.1132	0.06629
	G-4	0.8609	-	0.0344	0.02817
Modified Schwartz and Sinclair formula	G-0	0.53761	1	0.1583	0.10094
	G-1	0.80368	1	0.0851	0.03324
	G-2	0.81366	0.97	0.0509	0.02559
	G-3	0.64477	1	0.1132	0.07118
	G-4	0.8465	1	0.0344	0.03110

Influence of biodegradable polymeric coated slow-release granular urea on soil chemical properties, spinach yield and N uptake.

**Table 3:** Mean ± SE (n = 4) of initial (before treatment application) and final (after last harvest of spinach crop) soil characteristics, such as pH, electrical conductivity (EC), total organic carbon (TOC), dissolved organic carbon (DOC), mineral N (Nmin), plant available phosphorous (PAP) and potassium (PAK) of different treatments.

Sampling Occasion	Treatment	pH −	EC (dS m^−1^)	TOC (Mg ha^−1^)	DOC	Nmin	PAP	PAK
					(kg ha^−1^)			
**Initial**	Control	8.1 ± 0.06 ^a^*	0.19 ± 0.03 ^NS^**	3.9 ± 0.3 ^c^	11.1 ± 0.3 ^c^	9.2 ± 0.5 ^d^	7.6 ± 0.3 ^c^	295 ± 12 ^b^
**Final**	C	8.0 ± 0.29 ^a^	0.20 ± 0.01	4.7 ± 0.4 ^bc^	12.3 ± 0.4 ^bc^	10.7 ± 0.5 ^d^	8.8 ± 0.6 ^bc^	316 ± 8 ^ab^
	G-0	7.9 ± 0.01 ^a^	0.21 ± 0.01	5.0 ± 0.2 ^b^	13.6 ± 0.2 ^b^	19.4 ± 1.1 ^c^	9.1 ± 0.6 ^ab^	332 ± 17 ^ab^
	G-1	7.4 ± 0.05 ^b^	0.21 ± 0.02	6.1 ± 0.5 ^a^	16.2 ± 0.5 ^a^	23.2 ± 0.6 ^a^	9.8 ± 0.5 ^ab^	334 ± 25 ^ab^
	G-2	7.2 ± 0.03 ^b^	0.22 ± 0.01	6.1 ± 0.4 ^a^	16.5 ± 1.0 ^a^	24.2 ± 0.6 ^a^	10.5 ± 0.4 ^a^	357 ± 15 ^a^
	G-3	7.4 ± 0.06 ^b^	0.21 ± 0.02	5.6 ± 0.4 ^ab^	15.5 ± 0.4 ^a^	22.6 ± 0.4 ^ab^	9.6 ± 0.6 ^ab^	349 ± 9 ^a^
	G-4	7.4 ± 0.06 ^b^	0.21 ± 0.04	5.6 ± 0.3 ^ab^	15.1 ± 0.6 ^a^	20.7 ± 0.8 ^bc^	9.1 ± 0.5 ^ab^	336 ± 23 ^ab^

^*^ Values present in column followed by different letters as superscript are significantly different from each other (*p* ≤ 0.05); ** NS = non-significant.

## Nomenclature

**Symbols**	
ANR	Apparent nitrogen recovery
SEM	Scanning electron microscope
UV–VIS	Ultraviolet visible (UV) spectroscopy
FTIR	Fourier transform infrared
XRD	X-ray diffraction
FBC	Fluidized bed coater
G-0	Uncoated urea
G-1	10% starch + 5% polyvinyl alcohol (PVA) + 5% molasses
G-2	10% starch + 5% PVA + 5% paraffin wax
G-3	5% gelatin + 10% gum arabic + 5% PW
G-4	5% molasses + 5% gelatin + 10% gum arabic
C	Control (untreated soil)
EC	Electrical conductivity (EC)
DOC	Dissolved organic carbon
